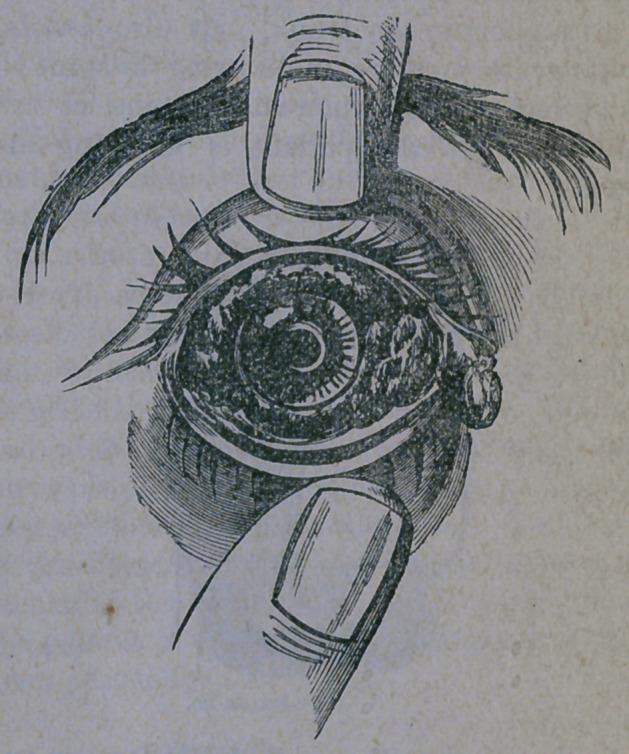# Purulent Ophthalmia

**Published:** 1873-10

**Authors:** 


### PURULENT OPHTHALMIA.

We now come to the consideration of an inflammation of the eye, more serious and destructive in its character, and more rapid in its development, than any which we have heretofore described in this series of articles. Scarcely a day passes that does not bring us for examination, a child rendered completely and hopelessly blind through the ravages of this fearful disease. Parents are so inclined to experiment with poultices, patent eye-waters, and the usual recommendations of officious acquaintances, when inflammation attacks the eyes of one of their children, with the hope that the disease is simply a "cold in the eye," that will perhaps yield to "home-made treatment," that much valuable time is lost, which should have been afforded the skillful specialist who could perhaps have arrested the disease and spared the patient from the serious consequences entailed by the ravages of this disease.

Purulent ophthalmia, although a disease most common to infants and children, is by no means confined to them, as older people frequently become victims to it. If the person is old enough to describe his symptoms, he will complain of great heat and itching in the lids, with a feeling as though some foreign body, as sand or dust, had lodged under the lids. Hot tears flow in abundance, and feel as though they must scald the cheek. The lids puff, become swollen and livid, and soon flakes of matter will be detected, mixed with the tears. The eyes become intolerant of the light, and the patient buries his face in a pillow or seeks a dark room. Speedily the disease develops until the lids become enormously swollen, the upper one hanging down over the under in a thick, heavy fold, discharging great quantities of thick, yellow matter.—The appearance of the lids at this stage of the disease is aptly illustrated in the cut here given :

As the disease progresses, the discharge of matter, swelling of the lids, and sensitiveness to light increase ; the patient sneezes at every admission of light to the room, and, from the constant burrowing of the face in the pillow, pimples are formed upon the face, rendering it sore and inflamed.

This immense swelling of the lids produces a constant and dangerous pressure upon the *cornea*, or clear portion of the eye, which, in combination with the irritating matter confined between the lids, soon involves the eye itself in the difficulty. The vessels of the cornea become so much compressed that the nutrition to that structure is cut off, subjecting it to ulceration and in time, consequent destruction. If an attempt is made to examine the condition of the cornea, by separating the lids, immense quantities of yellow, bloody matter will be discharged from between them, and the *conjunctiva*, (lining membrane) will roll out in great, livid folds, while the cornea will be found covered with matter and sunken deep into the swollen and inflamed membrane surrounding it, presenting the appearance below.

Having carefully washed the cornea free from matter, by means of a camel's hair brush and warm water, it can be examined, and should it present a cloudy or hazy appearance, evidence of ulceration is established, when no time should be lost in seeking treatment at the hands of a competent specialist. Eye waters, especially those containing *"sugar of lead," will bring certain destruction to an eye in this condition.* Nothing but constant care and watchfulness upon the part of the attending physician will save the eye from total destruction, after the cornea once becomes involved in the trouble.

The symptoms, aside from actual observation and scrutiny of the cornea itself, that will indicate the commencement of ulceration, are great pain in the brow, which abates for an hour or less, and then returns ; is worse towards night, and then extends to the side of the head. The sneezing and extreme sensitiveness to light are also indications of the commencement of ulceration in the cornea. If this ulceration is allowed to continue or is unskillfully treated, the cornea ruptures, when an escape of a portion of the humors of the eye occurs, all pain ceases, and the inflammation begins to subside, perhaps entirely disappearing within a few weeks.

The injury done the eye depends upon the extent of the ulcer. Should it cover the entire cornea, the patient is likely to be hopelessly blind. Should one-half, or less of it be involved, the resulting opacity will of course not be so great, and a possibility may exist of partially restoring vision by means of an operation for artificial pupil, a proceeding heretofore described in these articles. But we think our readers will agree with us in the opinion that much the best plan is to submit all such diseases of the eye to a competent physician, and not trust to the nostrums of the drug shop, or the meddlesome interference of kind but thoughtless friends. Nearly all the cases of blindness with which we come in contact could have been averted had the parties submitted themselves to the proper treatment early enough, and before they had exhausted the skill (?) and advice of persons who should have been kicked for offering it.

We hope we have rendered a sufficiently plain description of *purulent ophthalmia*, so that it can be readily recognized, and the proper means for arresting the disease sought, before complete destruction of vision ensues.

## Figures and Tables

**Figure f1:**
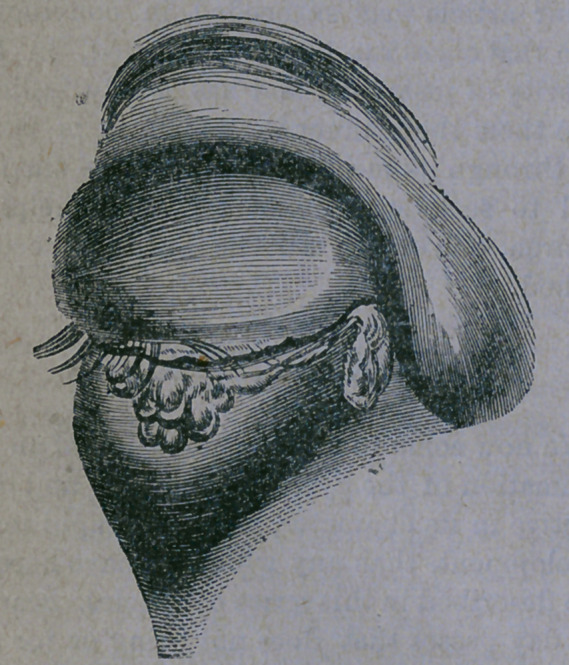


**Figure f2:**